# Application of Surface Stress-Driven Model for Higher Vibration Modes of Functionally Graded Nanobeams

**DOI:** 10.3390/nano14040350

**Published:** 2024-02-12

**Authors:** Giuseppe Lovisi, Luciano Feo, Annavirginia Lambiase, Rosa Penna

**Affiliations:** Department of Civil Engineering, University of Salerno, 84084 Fisciano, Italy; lfeo@unisa.it (L.F.); annlambiase@unisa.it (A.L.); rpenna@unisa.it (R.P.)

**Keywords:** functionally graded materials, Bernoulli–Euler nanobeams, surface stress-driven nonlocal model, free vibration analysis, surface energy effects, higher vibration modes

## Abstract

This paper employs a surface stress-driven nonlocal theory to investigate the synergistic impact of long-range interaction and surface energy on higher vibration modes of Bernoulli–Euler nanobeams made of functionally graded material. It takes into account surface effects such as the surface modulus of elasticity, residual surface stresses, surface density, and rotary inertia. The governing equation is derived through the application of Hamilton’s principle. The novelty of this work lies in its pioneering approach to studying higher-order vibrations, carefully considering the combination of long-range interactions and surface energy in nanobeams of functionally graded materials through a well-posed mathematical model of nonlocal elasticity. This study conducts a parametric investigation, examining the effects of the nonlocal parameter and the material gradient index for four static schemes: Cantilever, Simply-Supported, Clamped-Pinned and Clamped-Clamped nanobeams. The outcomes are presented and discussed, highlighting the normalized nonlocal natural frequencies for the second through fifth modes of vibration in each case under study. In particular, this study illustrates the central role of surface effects in the dynamic response of nanobeams, emphasizing the importance of considering them. Furthermore, the parametric analysis reveals that the dynamic response is influenced by the combined effects of the nonlocal parameter, the material gradient index, the shapes of the cross-sections considered, as well as the static scheme analyzed.

## 1. Introduction

Recent decades have seen significant progress in the field of nanoscience and nanotechnology, leading the scientific community to focus extensively on the analysis, modelling, and development of nanostructures [1,2,3]. Nanostructures are now employed in various fields and it is crucial to have accurate models for their reliable and efficient design.

Major challenges have been faced in the field of structural engineering that have led to the research and development of composite materials with the addition of nanoparticles and techniques for the study and prediction of static and dynamic structural response [4,5,6]. These challenges have stimulated innovation, leading to ever more advanced solutions and the optimization of structural performance. This reflects an ongoing commitment to overcoming obstacles and improving the resilience and efficiency of modern construction.

Further progress has been made with the introduction of a new class of composite materials, namely functionally graded (FG) materials, in the field of both structures and nanostructures, which allow high performance to be maintained even under severe thermal and mechanical stress [7,8,9,10,11,12].

Unlike structures at the macroscale, understanding the behavior of nanostructures in relation to their dimensions is crucial, given their extensive application in nanomechanical devices such as nanoelectromechanical actuators and nanomechanical resonators [13,14,15,16].

As commonly recognized, when the size of a structure reduces to the nanoscale, small-scale phenomena, negligible at the macroscale, become predominant. In particular, atomic interaction and surface effects play a crucial role that cannot be neglected at the nanoscale.

Various approaches exist for the study of nanostructures, including experimental investigations and molecular dynamics simulations [17,18]. Both are characterized by high computational costs and long analysis times.

In recent years, researchers have explored the introduction of non-classical continuum models for the study of nanostructures, appropriately modified to capture long-range interactions and surface effects.

One of the earliest non-classical continuum models is the Eringen [19] one, which differs from the classical continuum formulation by assuming that the stress at a point also depends on the deformation of the surrounding points. Eringen proposed a theory to capture this effect, where the stress field is obtained through an integral convolution, driven by strain, between the elastic strain field and an averaging kernel. To overcome the mathematical difficulties of integral resolution, Eringen later proposed the equivalent differential formulation (EDM) [20]. Additional nonlocal models have been developed from this formulation, including the nonlocal Eringen mixture model [21] and the nonlocal Lim gradient strain model [22], obtained by coupling the EDM model with the Mindlin gradient model [23].

In addition, Gurtin and Murdoch [24,25] introduced Surface Elasticity Theory (SET) to address the effects of surface energy. In this theory, the surface layer is considered as a membrane of negligible thickness, perfectly adhering to the mass continuum, and is characterized by unique properties and constitutive laws distinct from those that govern the bulk. This theory has often been coupled with the Eringen model to capture not only nonlocal effects but also surface effects.

Although these models have been widely used to study the static and dynamic aspects of nanostructures [26,27,28], the scientific community considers these models inapplicable for the study of nanostructures whose results are known as nanomechanics paradoxes [29,30,31,32].

To overcome the mathematical inconsistencies of the aforementioned models, Ro-mano and Barretta proposed a new Stress-Driven Model (SDM) of nonlocal elasticity [33], in which the integral convolution is a function of the stress field instead of the strain one. It has been extensively used in recent years to study both the static and the dynamic response of functionally graded nanobeams subjected to thermo-mechanical stresses [34,35,36,37,38,39,40,41,42].

Furthermore, Penna [43] recently extended the SDM model by coupling it with the SET to create the Surface Stress-Driven Model (SSDM). This model, well-posed mathematically, not only captures long-range interactions but also addresses surface effects. This new model has been recently used to investigate the free vibrations of functionally graded nanobeams [44], analyze static response in the presence of discontinuous loads [45], and investigate the effects of cracks in FG nanobeams [46].

The main innovation of this manuscript lies in the pioneering use of the SSDM to determine the frequencies of the higher vibration modes of FG nanobeams, due to its well-posed mathematical foundation and its consistent approach to the analysis of the structural response of nanostructures.

Specifically, it explores the effects of the nonlocal parameter, surface energy, and material gradient index on the natural frequency of the FG nanobeam, focusing on higher vibration modes for both rectangular and circular cross-sectional shapes.

This document’s structure as follows: in Section 2, the problem formulation is provided, including kinematics, geometry, material, and the governing equations of free oscillations derived from the use of Hamilton’s principle. A brief description of the SSDM and the size-dependent governing equations of transverse free oscillations are presented in Section 3. In the parametric analysis outlined in Section 4, we investigate and discuss the combined influences of the nonlocal parameter, surface effects, and gradient index on the higher-order vibration modes of the four static schemes considered. Finally, in Section 5, some concluding remarks are provided.

## 2. Problem Formulation

**Figure 1:**
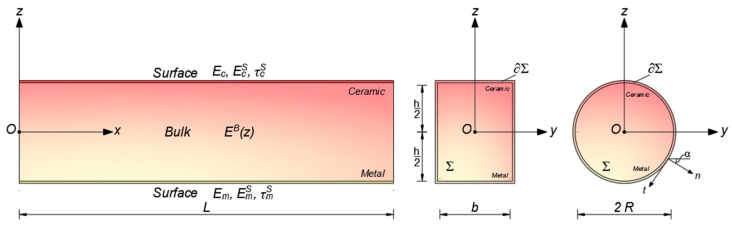
Coordinate system and configuration of the FG nanobeam: bulk continuum and surface layer.

Figure 1 shows the coordinate system and configuration of the FG nanobeam under investigation, composed of a bulk volume (*B*), made of a mixture of metal (*m*) and ceramic (*c*), and a thin surface layer (*S*), perfectly adhered to the bulk continuum (refer to Figure 1) with two distinct cross-sectional shapes.

As it is well-known, for a Bernoulli–Euler FG nanobeam whose mechanical and physical properties vary along the thickness (*z*), it can be assumed that the bulk elastic modulus of elasticity, $E^{B}=E^{B}\left(z\right)$, the surface modulus of elasticity, $E^{S}=E^{S}\left(z\right)$, the residual surface stress, $\tau^{S}=\tau^{S}\left(z\right)$, the bulk mass density, $\rho^{B}=\rho^{B}\left(z\right)$, and the surface mass density, $\rho^{S}=\rho^{S}\left(z\right)$, follow power-law functions as given below [28]

(1) $$E^{B}\left(z\right)=\;E_{m}+\left(E_{c}-E_{m}\right){\left(\frac{1}{2}+\frac{z}{\zeta }\right)}^{n}$$

(2) $$E^{S}\left(z\right)=\;E_{m}^{S}+\left(E_{c}^{S}-E_{m}^{S}\right){\left(\frac{1}{2}+\frac{z}{\zeta }\right)}^{n}$$

(3) $$\tau^{S}\left(z\right)=\;\tau_{m}^{S}+\left(\tau_{c}^{S}-\tau_{m}^{S}\right){\left(\frac{1}{2}+\frac{z}{\zeta }\right)}^{n}$$

(4) $$\rho^{B}\left(z\right)=\;\rho_{m}+\left(\rho_{c}-\rho_{m}\right){\left(\frac{1}{2}+\frac{z}{\zeta }\right)}^{n}$$

(5) $$\rho^{S}\left(z\right)=\;\;\rho_{m}^{S}+\left(\rho_{c}^{S}\;-\rho_{m}^{S}\right){\left(\frac{1}{2}+\frac{z}{\zeta }\right)}^{n}$$

n is the material gradient index (n≥0); ζ = h, in the case of a rectangular cross-section, and ζ = 2R for a circular one. Poisson’s ratio is here assumed to be constant ($\nu^{B}=\nu^{S}=\nu$).

### 2.1. Kinematic

The Bernoulli–Euler beam theory considers the following displacement field

(6) $$u\left(x,t\right)=u_{x}\left(x,z,t\right){\hat{e}}_{x}+u_{z}\left(x,z,t\right){\hat{e}}_{z}$$

where ${\hat{e}}_{x}$ and ${\hat{e}}_{z}$ are, respectively, the unit vectors along *x*- and *z*-axes; $u_{x}\left(x,z,t\right)$ and $u_{z}\left(x,z,t\right)$ indicate the Cartesian components of the displacement field along x and z axes at time *t*, expressed as follows

(7) $$u_{x}\left(x,z,t\right)=-z\frac{𝜕w\left(x,t\right)}{𝜕x}$$

(8) $$u_{z}\left(x,z,t\right)=w\left(x,t\right)$$

w(x,t)=w is the transverse displacement of the geometric center *O* (at time *t*).

Within the assumptions of the small strain and displacement theory, the simplified Green–Lagrange strain tensor is

(9) $$E\approx \varepsilon =\varepsilon_{xx}\;{\hat{e}}_{x}{\hat{e}}_{x}$$

where

(10) $$\varepsilon_{xx}=\varepsilon_{xx}\left(x,z,t\right)=-z\frac{{𝜕}^{2}w\left(x,t\right)}{𝜕x^{2}}$$

$\frac{{𝜕}^{2}w\left(x,t\right)}{𝜕x^{2}}$ is the geometric bending curvature χ. 

### 2.2. Governing Equations

The use of Hamilton’s principle allows us to obtain the governing equation of the free vibrations problem [44]

(11) $$\frac{{𝜕}^{2}M}{𝜕x^{2}}+T^{S}\frac{{𝜕}^{2}w}{𝜕x^{2}}=\left(A_{\rho }^{B}+A_{\rho }^{S}\right)\frac{{𝜕}^{2}w}{𝜕t^{2}}-\left(I_{\rho }^{B}+I_{\rho }^{S}\right)\frac{{𝜕}^{4}w}{𝜕x^{2}𝜕t^{2}}$$

where

(12) $$\left\{A_{\rho }^{B},I_{\rho }^{B}\right\}=\int_{\Sigma }\rho^{B}\left\{1,z^{2}\right\}d\Sigma$$

(13) $$\left\{A_{\rho }^{S},I_{\rho }^{S}\right\}=\oint_{𝜕\Sigma }\rho^{S}\left\{1,z^{2}\right\}d\sigma \;$$

(14) $${Τ}^{S}=\{\begin{array}{c} \underset{𝜕\Sigma }{\oint }\tau^{S}d\sigma \;\;\;\;\;\;\;\left(rectangular\;cross-section\right)\\ \underset{𝜕\Sigma }{\oint }\tau^{S}n_{z}d\sigma \;\;\;\;\;\;\;\;\;\;\;\left(circular\;cross-section\right) \end{array}$$

$n_{z}\;\mathrm{is}$ the z-component of the unit normal vector n, which is the outward normal to the cross-section lateral surface [43].

The appropriate boundary conditions of the FG nanobeam (at the nanobeam ends x=0,L) can be determined by selecting a single condition from each of the two pairs of Standard Boundary Conditions (SBCs) [44]

(15) $${\left[w\right]}_{0,L}\;\mathrm{or}\;{\left[\frac{𝜕M}{𝜕x}+T^{S}\frac{𝜕w}{𝜕x}+\left(I_{\rho }^{B}+I_{\rho }^{S}\right)\frac{{𝜕}^{3}w}{𝜕x𝜕t^{2}}\right]}_{0,L}$$

(16) $${\left[\frac{𝜕w}{𝜕x}\right]}_{0,L}\;\mathrm{or}\;{\left[M\right]}_{0,L}$$

M is the bending moment of FG nanobeam.

## 3. Surface Stress-Driven Model for Free Vibrations Analysis 

### 3.1. A Brief Outline of the Surface Stress-Driven Nonlocal Model

In this section, we provide a brief review of the surface stress-driven nonlocal model (SSDM) as outlined in [43]. Assuming a purely elastic constitutive behavior, the formulation of the surface stress-driven nonlocal model involves defining the bending curvature, χ, through the integral convolution, as detailed in the same reference [43]

(17) $$\chi =\overset{L}{\underset{0}{\int }}\Phi_{L_{c}}\left(x-\xi ,L_{c}\right)\left(-\frac{\;M^{*}}{I_{E}^{*}}\right)d\xi$$

where x and ξ are the positions of points of the domain of the Euclidean space occupied by the FG nanobeam at time *t*; $\Phi_{L_{c}}$ is an averaging kernel depending on the characteristic length of material, $L_{c}=\lambda_{c}L$; $I_{E}^{*}$ and M* are, respectively, the equivalent (bulk and surface) bending stiffness and the applied bending moment, defined as

(18) $$I_{E}^{*}=I_{E}^{B}+I_{E}^{S}=\underset{\Sigma }{\int }\left[E^{B}+\nu \;C\right]\;z^{2}d\Sigma +\underset{𝜕\Sigma }{\oint }E^{S}z^{2}d\sigma$$

(19) $$M^{*}=M^{*}\left(x,t\right)=M-M^{\tau }-\Lambda \frac{{𝜕}^{2}w}{𝜕t^{2}}$$

being

(20) $$C=\frac{2}{h}\left[{\left(\frac{z}{h}\right)}^{2}-\frac{3}{4}\right]\left(\tau_{c}^{S}+\tau_{m}^{S}\right)-\frac{1}{2z}\left(\tau_{c}^{S}-\tau_{m}^{S}\right)$$

(21) $$M^{\tau }=\oint_{𝜕\Sigma }\tau^{S}zd\sigma$$

(22) $$\Lambda =\int_{\Sigma }\nu \;D\;z\;d\Sigma$$

and

(23) $$D=2\frac{z}{h}\left[{\left(\frac{z}{h}\right)}^{2}-\frac{3}{4}\right]\left(\rho_{c}^{S}+\rho_{m}^{S}\right)-\frac{1}{2}\left(\rho_{c}^{S}+\rho_{m}^{S}\right)$$

As widely recognized, a specific function kernel, denoted as $\Phi_{L_{c}}$, is chosen to be

(24) $$\Phi_{L_{c}}\left(x,L_{c}\right)=\frac{1}{2L_{c}}\exp\left(-\frac{\left|x\right|}{L_{c}}\right)$$

for smooth source fields $\left(-\frac{\;M^{*}}{I_{E}^{*}}\right)$ in the domain [0,L]; the elastic curvature χ, as expressed in Equation (17), is equivalent to the following second-order differential equations, as outlined in [43]

(25) $$\left(1-L_{c}^{2}\frac{{𝜕}^{2}}{𝜕x^{2}}\right)\chi =-\frac{\;M^{*}}{I_{E}^{*}}$$

This equivalence is true if and only if the conventional Constitutive Boundary Conditions (CBCs) of the stress-driven nonlocal theory are satisfied at the ends of the FG nanobeam

(26) $$\frac{𝜕\chi \left(0\right)}{𝜕x}-\frac{1}{L_{c}}\chi \left(0\right)=0$$

(27) $$\frac{𝜕\chi \left(L\right)}{𝜕x}+\frac{1}{L_{c}}\chi \left(L\right)=0$$

By manipulating Equations (19) and (25), we can derive the expression for the resultant bending moment in the surface stress-driven nonlocal model

(28) $$M=M\left(x,t\right)=-\left(I_{E}^{B}+I_{E}^{S}\right)\frac{{𝜕}^{2}w}{𝜕x^{2}}+\left(I_{E}^{B}+I_{E}^{S}\right)L_{c}^{2}\frac{{𝜕}^{4}w}{𝜕x^{4}}+M^{\tau }+\Lambda \frac{{𝜕}^{2}w}{𝜕t^{2}}$$

### 3.2. Size-Dependent Governing Equation

By inserting Equation (28) into Equation (11), we obtain the equation that governs the dynamic problem of the FG nanobeam, incorporating both nonlocal and surface energy effects

(29) $$\begin{array}{cc} \left(I_{E}^{B}+I_{E}^{S}\right)L_{c}^{2}\frac{{𝜕}^{6}w}{𝜕x^{6}} & -\left(I_{E}^{B}+I_{E}^{S}\right)\frac{{𝜕}^{4}w}{𝜕x^{4}}+T^{S}\frac{{𝜕}^{2}w}{𝜕x^{2}}\\ & =\left(A_{\rho }^{B}+A_{\rho }^{S}\right)\frac{{𝜕}^{2}w}{𝜕t^{2}}-\Lambda \frac{{𝜕}^{4}w}{𝜕x^{2}𝜕t^{2}}-\left(I_{\rho }^{B}+I_{\rho }^{S}\right)\frac{{𝜕}^{4}w}{𝜕x^{2}𝜕t^{2}} \end{array}$$

with the corresponding standard (Equations (15) and (16)) and constitutive (Equations (26) and (27)) boundary conditions at the FG nanobeam ends (x=0,L).

Conclusively, by introducing the following dimensionless quantities

(30) $$\tilde{x}=\frac{x}{L}\;\tilde{w}=\frac{w}{L}\;\lambda_{c}=\frac{L_{c}}{L}\;\;\;\;\;\;\;\;\;{\tilde{M}}^{\tau }=\frac{M^{\tau }L}{I_{E}^{*}}\;{\tilde{T}}^{S}=\frac{{Τ}^{S}L^{2}}{I_{E}^{*}}\;{\tilde{A}}_{\rho }^{B}=\frac{A_{\rho }^{B}L^{4}}{I_{E}^{*}}\;{\tilde{A}}_{\rho }^{S}=\frac{A_{\rho }^{S}L^{4}}{I_{E}^{*}}\;{\tilde{I}}_{\rho }^{B}=\frac{I_{\rho }^{B}L^{2}}{I_{E}^{*}}\;{\tilde{I}}_{\rho }^{S}=\frac{I_{\rho }^{S}L^{2}}{I_{E}^{*}}\;\tilde{\Lambda }=\frac{1}{L^{2}}\;\frac{\Lambda }{A_{\rho }^{B}}\;{\tilde{g}}^{B}=\frac{1}{L^{2}}\;\frac{I_{\rho }^{B}}{A_{\rho }^{B}}\;{\tilde{g}}^{S}=\frac{1}{L^{2}}\;\frac{I_{\rho }^{S}}{A_{\rho }^{B}}\;\tilde{r}=\frac{A_{\rho }^{S}}{A_{\rho }^{B}}\;\tilde{\Lambda }=\frac{1}{L^{2}}\;\frac{\Lambda }{A_{\rho }^{B}}$$

and by using the classical method of separation variables, in which ω indicates the natural nonlocal frequency of transverse vibrations

(31) $$\tilde{w}\left(\tilde{x},t\right)=\tilde{W}\left(\tilde{x}\right)e^{i\omega t}$$

the dimensionless equation governing the linear transverse free vibrations based on SSDM can be expressed in terms of the non-dimensional spatial shape $\tilde{W}=\tilde{W}\left(\tilde{x}\right)$, as follows

(32) $$\lambda_{c}^{2}\frac{{𝜕}^{6}\tilde{W}}{𝜕{\tilde{x}}^{6}}-\frac{{𝜕}^{4}\tilde{W}}{𝜕{\tilde{x}}^{4}}+{\tilde{T}}^{S}\frac{{𝜕}^{2}\tilde{W}}{𝜕{\tilde{x}}^{2}}={\tilde{\omega }}^{2}\left(\left(\tilde{\Lambda }+{\tilde{g}}^{B}+{\tilde{g}}^{S}\right)\;\frac{{𝜕}^{2}\tilde{W}}{𝜕{\tilde{x}}^{2}}-\left(1+\tilde{r}\right)\tilde{W}\right)$$

being

(33) $${\tilde{\omega }}^{2}={\tilde{A}}_{\rho }^{B}\omega^{2}$$

with the corresponding dimensionless standard and constitutive boundary conditions

(34) $${\left[\tilde{W}\right]}_{\tilde{x}=0,1}\;\mathrm{or}\;{\left[\frac{𝜕\tilde{M}}{𝜕\tilde{x}}+{\tilde{T}}^{S}\frac{𝜕\tilde{W}}{𝜕\tilde{x}}+\left({\tilde{g}}^{B}+{\tilde{g}}^{s}\right)\frac{𝜕\tilde{W}}{𝜕\tilde{x}}\right]}_{\tilde{x}=0,1}$$

(35) $${\left[\frac{𝜕\tilde{W}}{𝜕\tilde{x}}\right]}_{\tilde{x}=0,1}\;\;\mathrm{or}\;{\left[\tilde{M}\right]}_{\tilde{x}=0,1}$$

(36) $$-\frac{{𝜕}^{3}\tilde{W}\left(0\right)}{𝜕{\tilde{x}}^{3}}+\frac{1}{\lambda_{c}}\frac{{𝜕}^{2}\tilde{W}\left(0\right)}{𝜕{\tilde{x}}^{2}}=0$$

(37) $$-\frac{{𝜕}^{3}\tilde{W}\left(1\right)}{\partial {\tilde{x}}^{3}}-\frac{1}{\lambda_{c}}\frac{{𝜕}^{2}\tilde{W}\left(1\right)}{\partial {\tilde{x}}^{2}}=0$$

$\tilde{M}$ is the dimensionless surface stress-driven nonlocal resultant moment expressed as follows

(38) $$\tilde{M}=\tilde{M}\left(\tilde{x}\right)=\lambda_{c}^{2}\frac{{𝜕}^{4}\tilde{W}\;}{𝜕{\tilde{x}}^{4}}-\frac{{𝜕}^{2}\tilde{W}\;}{𝜕{\tilde{x}}^{2}}+{\tilde{M}}^{\tau }-{\tilde{\omega }}^{2}\tilde{\Lambda }\;\tilde{W}$$

Equation (32) admits the following solution

(39) $$\tilde{W}=\overset{6}{\underset{k=1}{\sum }}q_{k}e^{\tilde{x}\;\beta_{k}}$$

It is essential to underline that the determination of the six unknown constants, indicated as $q_{k}$, depends on the satisfaction of the boundary conditions specified in Equations (34)–(37). 

To solve the above differential problem Equations (32)–(38), the authors have developed a Wolfram language code developed in Mathematica according to the procedure summarized in the flow chart of Box 1. The flow chart provides a visual representation of the process followed in solving the system of nonlinear equations, making it easier for readers to understand the methodology outlined in our scientific work.

Box 1 **Flow chart of the solution procedure of the nonlocal surface stress-driven model using the differential form.**

**STEP 1. Calculate the parameters**

Using the expressions (Equation (30)) defined in the manuscript to calculate $\tilde{\Lambda },\;{\tilde{g}}^{B},{\tilde{g}}^{S},\tilde{r},{\tilde{T}}^{S}$, and ${\tilde{M}}^{\tau }$.

**STEP 2. Solve the governing equation to get the expression of** $\tilde{W}$

Solve Equation (32) through the use of the “DSolve” function in Mathematica to get the expression of $\tilde{W}$ in terms of six integration constants *q_k_* to be determined.

**STEP 3. Set boundary conditions**

Define the boundary conditions for the examined static scheme by choosing from Equations (34)–(37).

**STEP 4. Flow chart for system solving:**

4.1 Initial iteration

- Set initial values or initial guesses for unknown variables (dimensionless nonlocal frequency). In our work, the frequencies obtained from the SDM model without surface effects were used as initial values.

4.2 Calculate determinant of coefficient matrix.

- Compute the determinant of the coefficient matrix.

4.3 Convergence check.

- Check the convergence of the iterative process.
- If convergent, proceed to the next section. Otherwise, update initial estimates.

4.4 Solve the system.

- Use the “FindRoot” function in Mathematica to find roots of the system of equations.

4.5 Final convergence verification.

- Recheck convergence and validity of obtained solutions.

4.6 Results.

- Analyze and interpret the obtained results.

## 4. Results and Discussion

In this paragraph, a higher-order free vibration analysis of Bernoulli–Euler FG nanobeams with length *L* = 10 nm is developed by considering Cantilever (C-F), Simply-Supported (S-S), Clamped-Pinned (C-P) and Doubly-Clamped (C-C) static configurations.

The analysis has been conducted using both the surface stress-driven model (SSDM) and the stress-driven model (SDM) without considering the surface energy effects. In addition, the present study encompasses two distinct cross-sectional shapes having the same second moment of area about their principal axis of geometric inertia *y*: a square cross-section (*b = h* = 0.1*L* = 1 nm) and a circular one of radius *R =* 0.571 nm.

The characteristic values of the physical and elastic properties of the two constituent materials, in terms of bulk Young’s modulus, $E_{c}^{B}$ and $E_{m}^{B}$, surface Young’s modulus, $E_{c}^{S}$ and $E_{m}^{S}$, residual surface stress, $\tau_{c}^{S}$ and $\tau_{m}^{S}$, bulk mass density, $\rho_{c}^{B}$ and $\rho_{m}^{B}$, and surface mass density, $\rho_{c}^{S}$ and $\rho_{m}^{S}$, are summarized in Table 1 [44]. 

The following results are expressed in terms of dimensionless normalized nonlocal frequency, obtained as the ratio between the nonlocal dimensionless frequency (Equation (31)) and the dimensionless local frequency ${\tilde{\omega }}_{loc}^{2}$. The dimensionless local frequency, ${\tilde{\omega }}_{loc}^{2}$, is the natural frequency of the first order (obtained by setting $\lambda_{c}={\tilde{g}}^{B}={\tilde{g}}^{S}=\tilde{\Lambda }=\tilde{r}={\tilde{T}}^{S}=n=0)$ and is assumed to be equal to 3.5160 for the Cantilever FG nanobeam, 9.8696 for the Simply-Supported, 15.4182 for the Clamped-Pinned, and 22.3733 for the Doubly-Clamped.

Firstly, in Table 2, Table 3, Table 4 and Table 5, the present approach has been validated by comparing the corresponding results, in terms of dimensionless nonlocal frequencies, to those obtained by Raimondo et al. in Ref. [41] for homogenous nanobeams by neglecting both the surface energy effects and the gyration radius $\left({\tilde{g}}^{B}=0\right)$.

Table 2, Table 3, Table 4 and Table 5 provide a summary of the results of the free vibration analysis in terms of normalized nonlocal high frequencies, corresponding to $\lambda_{c}\in \;${0.00^+^, 0.01, 0.02, 0.03, 0.04, 0.05, 0.06, 0.07, 0.08, 0.09, 0.10} and to *n* ∈{0,1,3} for the first five vibration modes. 

Looking at the results, it is evident that an increase in the material gradient index consistently leads to higher normalized nonlocal frequencies for the square cross-section, regardless of the boundary constraints considered. However, for the circular cross-section, the trend is conditioned by the specific static scheme considered.

**Figure 2:**
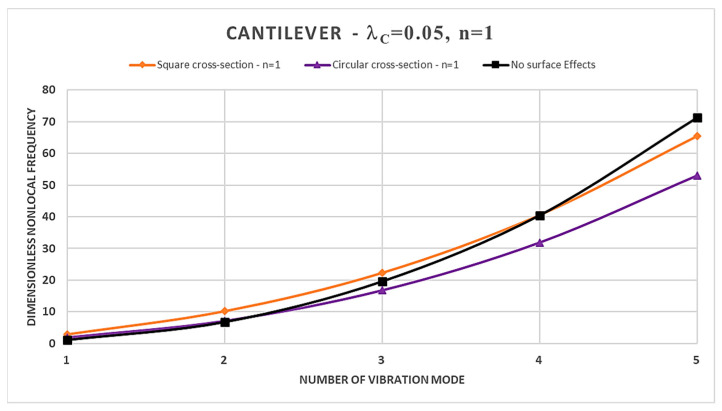
Dimensionless nonlocal frequencies of FG nanobeams vs. number of vibration modes evaluated for FG Cantilever (C-F) condition, with $\lambda_{c}=0.05$ and n=1.

**Figure 3:**
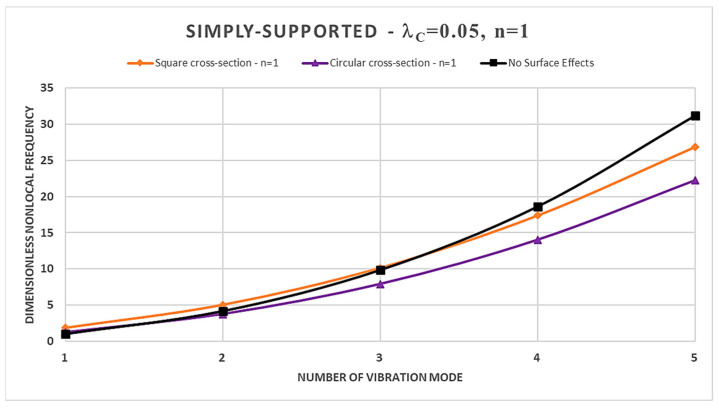
Dimensionless nonlocal frequencies of FG nanobeams vs. number of vibration modes evaluated for FG Simply-Supported (S-S) condition, with $\lambda_{c}=0.05$ and n=1.

**Figure 4:**
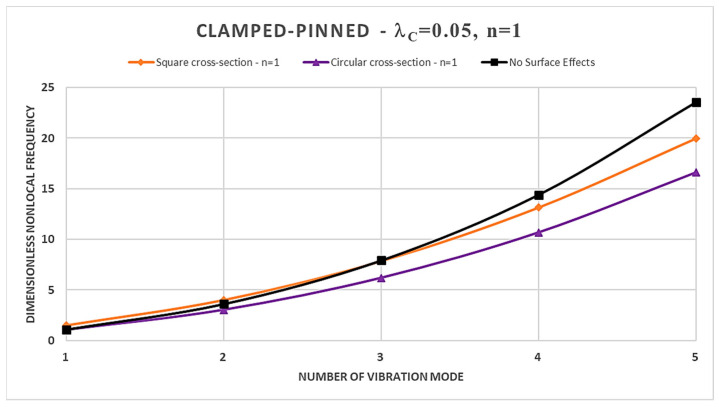
Dimensionless nonlocal frequencies of FG nanobeams vs. number of vibration modes evaluated for FG Clamped-Pinned (C-P) condition, with $\lambda_{c}=0.05$ and n=1.

**Figure 5:**
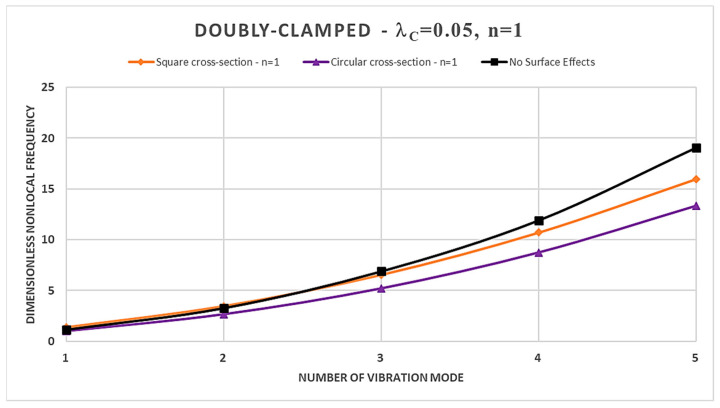
Dimensionless nonlocal frequencies of FG nanobeams vs. number of vibration modes evaluated for FG Doubly-Clamped (C-C) condition, with $\lambda_{c}=0.05$ and n=1.

Furthermore, from Table 2, Table 3, Table 4 and Table 5 and Figure 2, Figure 3, Figure 4 and Figure 5, it is easy to observe that the dimensionless nonlocal frequencies increase with increasing the order of the vibration modes for all the static schemes here considered. In addition, by fixing the values of the nonlocal parameter and the material gradient index, it is observed that the dimensionless nonlocal frequencies reach their maximum value in the case of the Cantilever FG nanobeam and the minimum one in the case of the Doubly-Clamped FG nanobeam for each vibration mode, regardless of the cross-sectional shapes chosen.

Therefore, it may be concluded that nonlocality strongly influences the normalized nonlocal frequencies, and its effects are stronger for higher vibration modes. In fact, increasing the nonlocal parameter always shows an increase in the dimensionless nonlocal frequencies.

Moreover, in the case of a square cross-section, the presence of surface effects results in additional stiffness, leading to an increase in the normalized nonlocal frequencies for the first three vibration modes compared to the model without surface effects in Ref. [41]; however, the surface energy causes a reduction in normalized nonlocal frequencies for the fourth and fifth vibration modes. On the contrary, FG nanobeams characterized by a circular cross-section show a more general dynamic response. In fact, it depends on the intertwined effects of the nonlocal parameter and the material gradient index, together with the boundary conditions at the nanobeam ends.

Finally, in Figure 2, Figure 3, Figure 4 and Figure 5, a comparison between the normalized nonlocal frequency curves for the surface stress-driven model (SSDM) and the stress-driven model (SDM) without surface effects is presented. The comparison spans all static configurations and the two types of cross-sections considered. For these illustrations, the parameters $\lambda_{c}=0.05$ and n=1 are set. As it can be observed, the SSDM consistently provides a stiffening behavior as the number of vibration modes increases. This trend is common in vibration systems, and Figure 2, Figure 3, Figure 4 and Figure 5 demonstrate how this behavior can be understood and described through the SSDM. Such a model not only highlights a common characteristic but also emphasizes how surface effects modify the frequency of higher-order vibration modes.

## 5. Conclusions

This study presents the main results of an application of the surface stress-driven model developed to investigate the coupled influences of the nonlocal parameter and the material gradient index on the higher-order free vibrations analysis of the functionally graded nanobeams.

The results have been successfully compared to those presented by Raimondo et al. in Ref. [41], where the surface energy effects were neglected, confirming the accuracy and reliability of the proposed approach.

The main conclusions are as follows:

- An increase in the material gradient index consistently results in an increase in the normalized nonlocal frequencies in the case of square cross-sections, regardless of whether the boundary constrains are considered; for the case of the circular cross-section, the trend is conditioned by the specific static scheme considered;
- The normalized nonlocal frequencies increase by increasing the order of the vibration modes for each static scheme considered;
- The dimensionless nonlocal frequencies reach their maximum value in the case of the C-F nanobeam and their minimum value in the case of the C-C nanobeam for each vibration mode, regardless of the cross-sectional shapes chosen;
- The nonlocality strongly influences the dimensionless frequencies, and its effects are stronger for higher vibration modes;
- By increasing the nonlocal parameter, the SSDM formulation always shows an increase in the normalized nonlocal frequencies;
- As the number of vibration modes increases, the SSDM always provides a stiffening behavior;
- In the case of a square cross-section, the presence of surface effects results in additional stiffness, leading to an increase in the dimensionless normalized nonlocal frequencies for the first three vibration modes compared to the model without surface effects; however, the surface energy causes a reduction in dimensionless nonlocal frequencies for the fourth and fifth vibration modes;
- The dynamic behavior of circular FG nanobeams is influenced by the coupled effects of the material gradient index and the nonlocal parameter, as well as by the boundary conditions at the nanobeams’ ends, and, therefore, it is not possible to define a specific trend;
- Finally, this study has provided valuable insights into the dynamic response of functionally graded nanobeams, shedding light on surface energy effects. However, it is imperative to acknowledge some limitations inherent in our research, as they play a crucial role in understanding the context and applicability of our findings. One limitation lies in the difficulty of comparing our results with those of experimental investigations; thus, we validated our model with numerical results from other authors. The comparison successfully demonstrated the validity of our approach and the results achieved in the present study have shown its ability to capture both nonlocal and surface energy effects in the higher-order dynamic response of functionally graded Bernoulli–Euler nanobeams. While these limitations temper the scope and generalizability of our findings, they also serve as a roadmap for future research. By openly acknowledging these constraints, we encourage subsequent researchers to build upon our work, addressing these limitations and expanding the horizons of knowledge in this field.

## Figures and Tables

**Table 1:** Physical and elastic properties of the two constituent materials of FG nanobeam.

Material	Parameters	Values	Unit
Ceramic (Si)	$E_{c}^{B}$	210	[GPa]
	$E_{c}^{S}$	−10.6543	[N/m]
	$\tau_{c}^{S}$	0.6048	[N/m]
	$\rho_{c}^{B}$	2370	[kg/m^3^]
	$\rho_{c}^{S}$	3.1688 × 10^−7^	[kg/m^2^]
Metal (Al)	$E_{m}^{B}$	70	[GPa]
	$E_{m}^{S}$	5.1882	[N/m]
	$\rho_{m}^{B}$	2700	[kg/m^3^]
	$\tau_{m}^{S}$	0.9108	[N/m]
	$\rho_{m}^{S}$	5.4610 × 10^−7^	[kg/m^2^]

**Table 2:** Dimensionless nonlocal frequencies of Cantilever (C-F) FG nanobeam for higher modes of vibration.

$\lambda_{c}$	Mode	No Surface Effects		Square Cross-Section			Circular Cross-Section		
		Present	Ref. [41]	n=0	n=1	n=3	n=0	n=1	n=3
0.00 ^+^	$1^{\mathrm{st}}$	1.0000	1.0000	2.2799	2.7626	3.0027	1.4981	1.7904	1.8865
	$2^{\mathrm{nd}}$	6.2669	6.2669	8.5112	9.7142	10.3436	5.9107	6.7299	6.9369
	$3^{\mathrm{th}}$	17.5475	17.5475	19.1051	20.4685	21.2408	13.9919	15.2841	15.4319
	$4^{\mathrm{th}}$	34.3860	-	33.8579	35.1898	35.9955	25.6689	27.4754	27.5037
	$5^{\mathrm{th}}$	56.8427	-	51.8652	53.0981	53.9124	40.5012	42.7677	42.6559
0.01	$1^{\mathrm{st}}$	1.0101	-	2.2946	2.7797	3.0209	1.5081	1.8020	1.8985
	$2^{\mathrm{nd}}$	6.3357	-	8.5816	9.7884	10.4201	5.9625	6.7859	6.9931
	$3^{\mathrm{th}}$	17.7713	-	19.3130	20.6764	21.4494	14.1515	15.4522	15.5981
	$4^{\mathrm{th}}$	34.9207	-	34.3280	35.6554	36.4594	26.0392	27.8616	27.8852
	$5^{\mathrm{th}}$	57.9402	-	52.7813	54.0042	54.8148	41.2392	43.5319	43.4113
0.03	$1^{\mathrm{st}}$	1.0309	-	2.3244	2.8137	3.0569	1.5284	1.8254	1.9228
	$2^{\mathrm{nd}}$	6.5093	-	8.7539	9.9674	10.6034	6.0897	6.9227	7.1298
	$3^{\mathrm{th}}$	18.5002	-	19.9871	21.3409	22.1109	14.6669	15.9929	16.1306
	$4^{\mathrm{th}}$	37.0797	-	36.2703	37.5626	38.3510	27.5482	29.4378	29.4370
	$5^{\mathrm{th}}$	63.0858	-	57.2474	58.4058	59.1879	44.7714	47.2065	45.1013
0.05	$1^{\mathrm{st}}$	1.0524	1.0524	2.3545	2.8477	3.0926	1.5490	1.8491	1.9471
	$2^{\mathrm{nd}}$	6.7278	6.7278	8.9646	10.1836	10.8232	6.2460	7.0897	7.2959
	$3^{\mathrm{th}}$	19.5634	19.5634	20.9672	22.2996	23.0608	15.4154	16.7767	16.9002
	$4^{\mathrm{th}}$	40.4580	-	39.3323	40.5640	41.3229	29.9179	31.9145	31.8738
	$5^{\mathrm{th}}$	71.3062	-	64.4541	65.5116	66.2479	50.4474	53.1200	52.8692
0.10	$1^{\mathrm{st}}$	1.1087	1.1087	2.4306	2.9331	3.1817	1.6011	1.9090	2.0088
	$2^{\mathrm{nd}}$	7.4325	7.4325	9.6210	10.8467	11.4935	6.7369	7.6095	7.8101
	$3^{\mathrm{th}}$	23.2560	23.2560	24.3703	25.6113	26.3313	18.0146	19.4950	19.5662
	$4^{\mathrm{th}}$	52.1914	-	50.0444	51.0811	51.7429	38.1909	40.5729	40.3977
	$5^{\mathrm{th}}$	99.0703	-	88.9574	89.7452	90.3647	69.7106	73.2203	72.7155

The symbol ^+^ means for the limit that tends to zero, the same applies to the following tables.

**Table 3:** Dimensionless nonlocal frequencies of Simply-Supported (S-S) FG nanobeam for higher modes of vibration.

$\lambda_{c}$	Mode	No Surface Effects		Square Cross-Section			Circular Cross-Section		
		Present	Ref. [41]	n=0	n=1	n=3	n=0	n=1	n=3
0.00 ^+^	$1^{\mathrm{st}}$	1.0000	1.0000	1.5375	1.8218	1.9718	1.0416	1.2124	1.2649
	$2^{\mathrm{nd}}$	4.0000	4.0000	4.4988	4.8850	5.1033	3.2633	3.5906	3.6395
	$3^{\mathrm{th}}$	9.0000	9.0000	9.0110	9.4167	9.6589	6.7903	7.2932	7.3111
	$4^{\mathrm{th}}$	16.0000	-	14.7940	15.1850	15.4386	11.4929	12.1644	12.1399
	$5^{\mathrm{th}}$	24.9999	-	21.5186	21.8782	22.1411	17.1945	17.9903	17.9198
0.01	$1^{\mathrm{st}}$	1.0005	-	1.5379	1.8221	1.9721	1.0419	1.2127	1.2651
	$2^{\mathrm{nd}}$	4.0077	-	4.5052	4.8909	5.1090	3.2684	3.5953	3.6445
	$3^{\mathrm{th}}$	9.0391	-	9.0449	9.4485	9.6905	6.8170	7.3206	7.3380
	$4^{\mathrm{th}}$	16.1233	-	14.8988	15.2869	15.5391	11.5764	12.2508	12.2248
	$5^{\mathrm{th}}$	25.3003	-	21.7635	22.1185	22.3792	17.3934	18.2228	18.1220
0.03	1^st^	1.0042	-	1.5402	1.8240	1.9739	1.0438	1.2146	1.2669
	$2^{\mathrm{nd}}$	4.0662	-	4.5541	4.9359	5.1521	3.3068	3.6349	3.6824
	$3^{\mathrm{th}}$	9.3321	-	9.2994	9.6921	9.9285	7.0167	7.5272	7.5399
	$4^{\mathrm{th}}$	17.0355	-	15.6762	16.0440	16.2860	12.1951	12.8912	12.8545
	$5^{\mathrm{th}}$	27.4868	-	23.5495	23.8728	24.1196	18.8430	19.6904	19.5969
0.05	$1^{\mathrm{st}}$	1.0110	1.0110	1.5446	1.8278	1.9774	1.0474	1.2181	1.2702
	$2^{\mathrm{nd}}$	4.1740	4.1740	4.6445	5.0194	5.2322	3.3779	3.7440	3.7525
	$3^{\mathrm{th}}$	9.8598	9.8598	9.7601	10.1345	10.3614	7.3780	7.9012	7.9060
	$4^{\mathrm{th}}$	18.6338	-	17.0446	17.3809	17.6073	13.2832	14.0186	13.9640
	$5^{\mathrm{th}}$	31.2018	-	26.5951	26.8736	27.1016	21.3127	22.2403	22.1147
0.10	$1^{\mathrm{st}}$	1.0389	1.0389	1.5628	1.8431	1.9916	1.0623	1.2326	1.2838
	$2^{\mathrm{nd}}$	4.5952	4.5952	5.0033	5.3532	5.5536	3.6588	3.9955	4.0315
	3^th^	11.8266	11.8266	11.4990	11.8171	12.0146	8.7368	9.3128	9.2904
	$4^{\mathrm{th}}$	24.3000	-	21.9395	22.1933	22.3809	17.1639	18.0516	17.9408
	$5^{\mathrm{th}}$	43.7693	-	36.9613	37.1361	37.3285	29.7045	30.9210	30.6978

The symbol ^+^ means for the limit that tends to zero, the same applies to the following tables.

**Table 4:** Dimensionless nonlocal frequencies of Clamped-Pinned (C-P) FG nanobeam for higher modes of vibration.

$\lambda_{c}$	Mode	No Surface Effects		Square Cross-Section			Circular Cross-Section		
		Present	Ref. [41]	n=0	n=1	n=3	n=0	n=1	n=3
0.00 ^+^	$1^{\mathrm{st}}$	1.0000	1.0000	1.2776	1.4411	1.5296	0.8955	1.0101	1.0362
	$2^{\mathrm{nd}}$	3.2406	3.2406	3.4794	3.7020	3.8302	2.5552	2.7816	2.8020
	$3^{\mathrm{th}}$	6.7614	6.7614	6.6373	6.8740	7.0185	5.0325	5.3803	5.3797
	$4^{\mathrm{th}}$	11.5623	-	10.5625	10.7930	10.9466	8.2399	8.6987	8.6705
	$5^{\mathrm{th}}$	17.6435	-	15.0470	15.2594	15.4214	12.0630	12.5991	12.5415
0.01	$1^{\mathrm{st}}$	1.0108	-	1.2883	1.4520	1.5407	0.9035	1.0186	1.0447
	$2^{\mathrm{nd}}$	3.2813	-	3.5167	3.7389	3.8670	2.5840	2.8118	2.8318
	$3^{\mathrm{th}}$	6.8644	-	6.7266	6.9619	7.1059	5.1033	5.4539	5.4523
	$4^{\mathrm{th}}$	11.7807	-	10.7415	10.9692	11.1219	8.3852	8.8487	8.8187
	$5^{\mathrm{th}}$	18.0579	-	15.3677	15.5760	15.7366	12.3295	12.8723	12.8117
0.03	$1^{\mathrm{st}}$	1.0375	-	1.3129	1.4763	1.5651	0.9221	1.0381	1.0639
	$2^{\mathrm{nd}}$	3.4104	-	3.6320	3.8504	3.9766	2.6734	2.9048	2.9230
	$3^{\mathrm{th}}$	7.2715	-	7.0827	7.3084	7.4476	5.3834	5.7448	5.7382
	$4^{\mathrm{th}}$	12.7887	-	11.5928	11.8040	11.9489	9.0666	9.5542	9.5140
	$5^{\mathrm{th}}$	20.1774	-	17.0764	17.2602	17.4109	13.8938	14.3097	14.2316
0.05	$1^{\mathrm{st}}$	1.0703	1.0703	1.3416	1.5041	1.5925	0.9442	1.0611	1.0864
	$2^{\mathrm{nd}}$	3.5967	3.5967	3.7972	4.0084	4.1311	2.8017	3.0378	3.0528
	$3^{\mathrm{th}}$	7.9066	7.9066	7.6426	7.8527	7.9839	5.8222	6.2008	6.1861
	$4^{\mathrm{th}}$	14.4028	-	12.9725	13.1593	13.2927	10.1653	10.6941	10.6379
	$5^{\mathrm{th}}$	23.5687	-	19.8429	19.9935	20.1319	15.9729	16.6313	16.5260
0.10	$1^{\mathrm{st}}$	1.1749	1.1749	1.4304	1.5875	1.6737	1.0131	1.1318	1.1418
	$2^{\mathrm{nd}}$	4.2468	4.2468	4.3784	4.5650	4.6750	3.2517	3.5049	3.5094
	$3^{\mathrm{th}}$	10.1365	10.1365	9.6348	9.8008	9.9094	7.3762	7.8211	7.7804
	$4^{\mathrm{th}}$	19.9215	-	17.7410	17.8691	17.9770	13.9492	14.6302	14.5244
	$5^{\mathrm{th}}$	34.7602	-	29.0444	29.1236	29.2425	23.4328	24.3488	24.1620

The symbol ^+^ means for the limit that tends to zero, the same applies to the following tables.

**Table 5:** Dimensionless nonlocal frequencies of Doubly-Clamped (C-C) FG nanobeam for higher modes of vibration.

$\lambda_{c}$	Mode	No Surface Effects		Square Cross-Section			Circular Cross-Section		
		Present	Ref. [41]	n=0	n=1	n=3	n=0	n=1	n=3
0.00 ^+^	$1^{\mathrm{st}}$	1.0000	1.0000	1.1448	1.2397	1.2927	0.8225	0.9067	0.9181
	$2^{\mathrm{nd}}$	2.7565	2.7565	2.8670	3.0045	3.0849	2.1245	2.2952	2.3016
	$3^{\mathrm{th}}$	5.4039	5.4039	5.2236	5.3724	5.4652	3.9804	4.2402	4.2136
	$4^{\mathrm{th}}$	8.9329	-	8.0769	8.2231	8.3237	6.3237	6.6614	6.6334
	$5^{\mathrm{th}}$	13.3443	-	11.2860	11.4210	11.5289	9.0751	9.4637	9.4155
0.01	$1^{\mathrm{st}}$	1.0214	-	1.1656	1.2606	1.3137	0.8382	0.9233	0.9345
	$2^{\mathrm{nd}}$	2.8211	-	2.9263	3.0633	3.1436	2.1703	2.3433	2.3491
	$3^{\mathrm{th}}$	5.5464	-	5.3466	5.4941	5.5864	4.0783	4.3420	4.3320
	$4^{\mathrm{th}}$	9.2036	-	8.2957	8.4395	8.5393	6.5027	6.8459	6.8158
	$5^{\mathrm{th}}$	13.8141	-	11.6416	11.7729	11.8798	9.3734	9.7689	9.7176
0.03	$1^{\mathrm{st}}$	1.0726	-	1.2135	1.3075	1.3603	0.8745	0.9615	0.9720
	$2^{\mathrm{nd}}$	3.0062	-	3.0942	3.2271	3.3054	2.3001	2.4789	2.4823
	$3^{\mathrm{th}}$	6.0329	-	5.7732	5.9120	6.0001	4.4141	4.6914	4.6761
	$4^{\mathrm{th}}$	10.2718	-	9.1936	9.3233	9.4169	7.2234	7.5923	7.5524
	$5^{\mathrm{th}}$	15.8833	-	13.2967	13.4079	13.5077	10.5476	11.1652	11.0979
0.05	$1^{\mathrm{st}}$	1.1349	1.1349	1.2701	1.3620	1.4138	0.9178	1.0066	1.0161
	$2^{\mathrm{nd}}$	3.2614	3.2614	3.3252	3.4508	3.5253	2.4786	2.6649	2.6648
	$3^{\mathrm{th}}$	6.8814	6.8814	6.4143	6.5396	6.6208	4.9160	5.2143	5.1908
	$4^{\mathrm{th}}$	11.9129	-	10.5959	10.7059	10.7909	8.3411	8.7528	8.6978
	$5^{\mathrm{th}}$	19.0851	-	15.9012	15.9867	16.0780	12.8489	13.3540	13.2626
0.10	$1^{\mathrm{st}}$	1.1766	1.1766	1.4485	1.5316	1.5790	1.0549	1.1487	1.1546
	$2^{\mathrm{nd}}$	4.1322	4.1322	4.1225	4.2251	4.2875	3.0922	3.3059	3.2939
	$3^{\mathrm{th}}$	9.2325	9.2325	8.6431	8.7337	8.7977	6.6519	7.0286	6.9793
	$4^{\mathrm{th}}$	17.4074	-	15.3466	15.4132	15.4815	12.1120	12.6785	12.5778
	5^th^	29.4704	-	24.4213	24.4545	24.5363	19.7635	20.5082	20.3449

The symbol ^+^ means for the limit that tends to zero, the same applies to the following tables.

## Data Availability

Data are contained within the article.

## Nomenclature

E	Euclidean space	$\lambda_{c}$	Nonlocal parameter
*L*	Length of FG nanobeam	$\nu^{B},\;\nu^{S}$	Poisson’s ratios of the bulk and surface layer
Σ	Generic cross-section	*n*	material gradient index
∂Σ	Perimeter of Σ	$E_{c}$	Young’s modulus ceramic
{O,x,y, z}	Cartesian coordinate system	$E_{m}$	Young’s modulus metal
*O*	Geometric center of Σ	$\rho_{c}$	Mass density of ceramic
*x*	Axis of FG nanobeam	$\rho_{m}$	Mass density of metal
*y*, *z*	Principal axes of geometric inertia of Σ	$E_{c}^{S}$	Surface Young modulus of ceramic
*b*, *h*	Width and thickness Σ	$E_{m}^{S}$	Surface Young modulus of metal
*R*	Radius of Σ	$\rho_{c}^{S}$	Surface mass density of ceramic
*B*, *S*	Bulk and surface layers of FG material	$\rho_{m}^{S}$	Surface mass density of metal
*E*	Euclidean space	$\tau_{c}^{S}$	Residual surface stress of ceramic
*L*	Length of FG nanobeam	$\tau_{m}^{S}$	Residual surface stress of metal
